# Novel amphiphilic polyvinylpyrrolidone functionalized silicone particles as carrier for low-cost lipase immobilization

**DOI:** 10.1098/rsos.172368

**Published:** 2018-06-13

**Authors:** Shan Zhang, Qianchun Deng, Ya Li, Mingming Zheng, Chuyun Wan, Chang Zheng, Hu Tang, Fenghong Huang, Jie Shi

**Affiliations:** Hubei Key Laboratory of Lipid Chemistry and Nutrition, Oil Crops and Lipids Process Technology National and Local Joint Engineering Laboratory, Key Laboratory of Oilseeds Processing, Ministry of Agriculture, Oil Crops Research Institute, Chinese Academy of Agricultural Sciences, Wuhan 430062, People's Republic of China

**Keywords:** amphiphilic, polyvinylpyrrolidone, silicone, immobilization

## Abstract

The high catalytic activity, specificity and stability of immobilized lipase have been attracting great interest. How to reduce the cost of support materials has always been a hot topic in this field. Herein, for the development of low-cost immobilized lipase, we demonstrate an amphiphilic polyvinylpyrrolidone (PVP) grafted on silicone particle (SP) surface materials (SP-PVP) with a rational design based on interfacial activation and solution polymerization. Meanwhile, hydrophilic pristine SP and hydrophobic polystyrene-corded silicone particles (SP-Pst) were also prepared for lipase immobilization. SP-PVP was characterized by X-ray diffraction, scanning electron microscopy, X-ray photoelectron spectroscopy, Fourier transform infrared spectroscopy and thermogravimetry. Our results indicated that the lipase loading amount on the SP-PVP composites was about 215 mg of protein per gram. In the activity assay, the immobilized lipase SP-PVP@CRL exhibited higher catalysis activity and better thermostability and reusability than SP@CRL and SP-Pst@CRL. The immobilized lipase retained more than 54% of its initial activity after 10 times of re-use and approximately trended to a steady rate in the following cycles. By introducing the interesting amphiphilic polymer to this cheap and easily obtained SP surface, the relative performance of the immobilized lipase can be significantly improved, facilitating interactions between the low-cost support materials and lipase.

## 1. Introduction

The high effectiveness, high specificity, low cost and environment-friendly properties make biocatalysts the preferable alternative to the classic chemical modifications especially in the pharmaceutical chemistry and food modification with the rapid development of green and sustainable chemistry [1,2]. Lipases, as one of the most extensively used biocatalysts, have gained increased attention in a wide range of reactions such as esterification, hydrolysis and aminolysis due to their excellent chemo-, regio- and stereoselective properties [3]. Nevertheless, the practical industrial applications are often limited by a lack of long-term operational stability and high production costs resulting from the difficulties in recovery and recycling for reuse [4]. In recent years, numerous efforts have been devoted to tackle these issues for delivering reusable and stable enzymes [5–7]. Immobilizing lipase on some cheap and easily obtained supports in different ways can lead to the properties of enhanced performance, improved stability and prolonged reusability, hence reducing the cost of biocatalysts in enzyme application [8–10]. Various immobilization strategies such as multipoint or multisubunit are designed to improve the stability and activity of an enzyme [11–16]; also, several approaches try to use site-directed mutagenesis to directly improve enzyme performances and then immobilize the enzymes on a support [17].

A number of suggested lipase immobilization techniques [18], including covalent bonding [19], entrapment [20,21] and physical adsorption [22–25] with different supports, have assorted advantages and disadvantages to make a comparison difficult. Encapsulating lipase has the drawbacks of mass transfer limitations and low enzyme loading, while strong covalent attachment may cause lipase to be irreversibly deactivated to some extent [4]. On the other hand, the high activity, low cost and simple operation character make physical adsorption to be used widely [26,27]. Furthermore, as an important part for immobilization, chemically inert supports [28–30] prompt the multiple use of lipase by improving its physical, chemical and mechanical stability, as well as lipase lifetime [31]. Inorganic supports such as silica [32–34], titania (TiO_2_) [35,36], carbon nanotube [37–39], Fe_2_O_3_/Fe_3_O_4_ [40] and hydrotalcite (LDHs) [41] have been proved to be of great potential for lipase confinement [3]. For their large surface areas, low cost, inertness and stability at elevated temperatures, silicone particles (SP) have drawn great focus in enzyme immobilization. However, unfortunately, for lipase, the hydrophilicity of SP impedes the expression of the real activity, probably on account of an inadequate conformation of the lipase caused by interaction with silanol groups [42]. As we know, the lipases have dual nature as they are mostly hydrophilic with some hydrophobic patches surrounding their active centre that is often blocked by the polypeptide lid [43,44]. However, the lipases have a specific mechanism of action called interfacial activation [45,46]. According to reports, the lipase was inclined to an open-form structure in the presence of hydrophobic media or surface [47,48]. Yet, in actual fact, extremely hydrophobic carriers can cause higher lipase loading for protein molecular crowding, leading to lipase deactivation [49]. During the immobilization processes, protein loading and hydrophobicity/hydrophilicity of the carrier inevitably effect structural and dynamic feature changes in lipases [16]. Therefore, adjusting the properties of hydrophilicity and hydrophobicity of the SP surface by suitable functional groups can enable the SP-based materials to be an ideal carrier for the low-cost immobilization of lipase.

**Figure 1.**
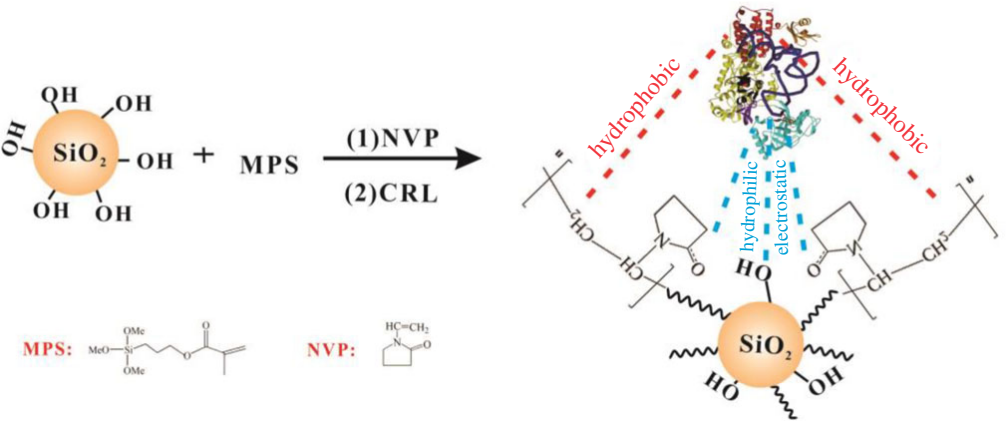
A schematic illustration of the preparation of SP-PVP@CRL.

Polyvinylpyrrolidone (PVP), due to its properties of hydrophilicity, lipophilicity, non-ionicity and non-toxicity, has popularly emerged in many areas especially in medicine, food and cosmetics, which are closely related to people's health. In this paper, PVP was selected as the surface modifier for its simple molecular structure, availability, amphiphilicity and safety. Upon modification by PVP, the surface characteristics of SP alternated from hydrophilic to amphiphilic for better interaction with the substrate and more durability to changes of the environment, namely by forming profitable reaction interfaces for lipase. Moreover, interaction between PVP and lipase via electrostatic and/or H-bond interactions [50] will give us an efficient immobilization process. The sequential steps for preparing SP-PVP@CRL are presented in figure 1; firstly, the silanol groups on the SP and the hydrophilic domains on the PVP interacted with *Candida rugosa* lipase (CRL) via electrostatic and/or H-bond interaction. Meanwhile, the hydrophobic units on the PVP interacted with lipase via hydrophobic interaction, which may be effective to keep a ‘lid open' conformation to expose the active sites for lipase; on the other hand, the advantageous properties of SP such as high specific surface area, high mechanical stability and safety are also beneficial to catalytic reaction and the immobilization of lipase.

On the basis of the foregoing, we demonstrated the concept that, by immobilizing lipase on the PVP-grafted SP via physical absorption, micro-reactors for lipase catalytic reaction could be formed in the substrate environment, which could increase the stability of lipase, improving the conformation of lipase to offer better catalytic performance. To investigate the interfacial behaviour between carriers and lipase, hydrophilic pristine SP and hydrophobic polystyrene-corded silicone particles (SP-Pst) were also prepared for lipase immobilization. CRL was selected here as a model enzyme in order to assess the potential applicability of PVP-grafted SP as a new carrier. The physical properties, relative activity, stability and reusability were all analysed in this work.

## 2. Material and methods

### 2.1. Materials

3-Methacryloxypropyltrimethoxysilane (MPS) was purchased from WD Silicone Co., Ltd (Wuhan, China); *N*-vinylpyrrolidone (NVP), ammonium persulfate, triethylamine, PVP-K30, styrene (st) and azobisisobutyronitrile (AIBN) were purchased from Sinopharm Chemical Reagent Co., Ltd (Shanghai, China) and used without further purification. SP were purchased from Ocean Chemical Engineering Co., Ltd (Qingdao, China). CRL (lyophilized powder, Type VII, 700 U/mg solid) and *p*-nitrophenyl palmitate (*p*-NPP) were purchased from Sigma-Aldrich (St Louis, USA). All other solvents or chemicals used were of analytic grade.

### 2.2. Preparation of polyvinylpyrrolidone-grafted silicone particle and polystyrene-grafted silicone particle

SP (30 g) were placed in 150 ml of methanesulfonic acid aqueous solution with a concentration of 5%, and then activated for 4 h under stirring at 102°C. After filtration, the mixture was washed with distilled water repeatedly and dried under vacuum for 24 h [51].

The activated SP were added into ethanol, and then a certain amount of MPS was added. Under the protection of nitrogen, the reaction was carried out for 24 h at 50°C. After the reaction was over, the sample was washed with ethanol repeatedly to remove excess MPS and then dried under vacuum to obtain finally MPS-SP materials [52,53].

PVP-K30 (4 g) was dispersed in 200 g ethanol/water mixture (94.5/5.5, w/w) and then 3 g of MPS-SP was added. The mixture was subjected to ultrasonic treatment for 30 min and then 2 g of styrene and 0.2 g of AIBN were added slowly. The reaction was carried out at 70°C under a N_2_ atmosphere for 12 h. After the termination of polymerization, the mixture was filtered and washed with ethanol more than five times [52]. Finally, the products were dried under vacuum to obtain a pale grey SP-Pst material.

Deionized water (140 ml), 2.5 g MPS-SP, 0.28 g initiator ammonium persulfate, 0.4 g activator triethylamine and 2.1 g NVP were added in a flask equipped with a N_2_ inlet and a water condenser. The reaction was carried out at 70°C under a N_2_ atmosphere for 8 h. After the termination of polymerization, the mixture was filtered and washed with ethanol several times. Finally, the products were dried under vacuum to obtain a pale brownish-yellow SP-PVP material.

### 2.3. Immobilization of lipase

The obtained SP-PVP and SP-Pst and original SP were dispersed in lipase solution, and then the mixed solution was incubated at 37°C in a shaker at 160 r.p.m. for 6 h. The immobilized lipases were washed with phosphate buffer (0.1 M, pH = 7.0) three times and freeze-dried in a vacuum chamber. The three obtained immobilized lipases, SP-PVP@CRL, SP-Pst @CRL and SP@CRL, were stored at 4°C until they were used.

### 2.4. Lipase activity assay

The protein concentration was determined by the Bradford method [54], using bovine serum albumin as the standard. The amount of immobilized enzyme was calculated by subtracting the amount of un-immobilized enzyme from the total lipase used for the immobilization. One unit of activity (U) was defined as the amount of enzyme that hydrolyses 1 µmol of *p*-NPP per minute under the conditions described previously.

The activities of the free and immobilized lipases were measured by hydrolysing *p*-NPP under 160 r.p.m. at 37°C for 5 min. The concentration of the hydrolysis product (*p*-nitrophenol (*p*-NP)) was measured using a spectrophotometric method at 410 nm [54]. The relative activities were defined by assuming the maximum activity value of the immobilized and free lipase under optimal conditions as 100%. The effects of pH and temperature on the activities of free and immobilized lipases were investigated under a variety of pHs and temperatures. Thermal stabilities of free and immobilized lipases were studied by measuring the residual activities of the lipase after incubation at 50°C for 0–4 h. The recyclability of the biocatalyst was evaluated in consecutive batches of the hydrolysis of *p*-NPP. Immobilized CRL (30 mg) and 1 ml 0.5% *p*-NPP ethanol solution (w/v) were mixed in 1 ml of 0.1 M (pH 7.0) phosphate buffer for 30 min at 37°C under mild stirring. The reusability of the immobilized lipase was determined by recovering the immobilized lipase and comparing with the first run (activity defined as 100%).

### 2.5. Characterization

Powder X-ray diffraction (XRD) patterns of the products were collected on a Bruker D8 ADVANCE X-ray diffractometer. The scanning electron microscopy (SEM) images were obtained with a Hitachi U8010 field emission scanning electron microscope. Fourier transform infrared (FTIR) spectra were recorded with a Bruker TENSOR27. The thermal stability of samples was studied with a thermogravimetry (TG) analyser (TAQ50, Netzsch, Germany) at a heating rate of 50°C min^–1^ in a nitrogen atmosphere.

## 3. Results and discussion

SP-PVP was prepared by a simple classical polymerization. SP was firstly reacted with methanesulfonic acid and MPS for surface activation to obtain MPS-PVP. Then, the PVP was grafted by polymerization, by which the precursor NVP, initiator ammonium persulfate and activator triethylamine reacted with the activated MPS-PVP.

**Figure 2.**
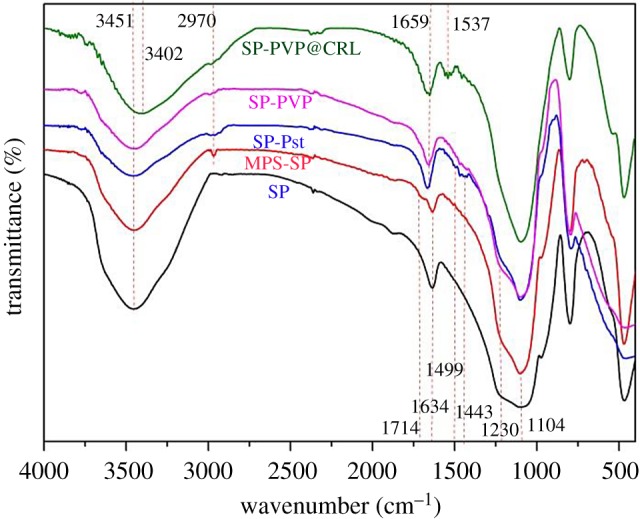
The FTIR spectra of SP, MPS-SP, SP-Pst, SP-PVP and SP-PVP@CRL.

Figure 2 shows the FTIR spectra of particles: pristine SP, MPS-SP, SP-Pst, SP-PVP and SP-PVP @CRL. For the pristine SP, the wide absorption band at 3451 cm^−1^ was assigned to silanol groups and trace water. After the surface modification of SP, all of the absorption at 3451 cm^−1^ was weakened, which might arise from the interaction between silanol groups and the modifier. When the MPS was introduced, new absorptions appeared at 2970 cm^−1^ and 1714 cm^−1^, which represented the asymmetric stretching vibration of C–H and the stretching vibration of the C = O bond. Namely, these results showed that MPS had been successfully modified on the SP surface (MPS-SP). After grafting of the Pst on the SP, the framework vibration absorptions of the benzene ring at 1499 and 1443 cm^−1^ were noted. In addition, when the PVP was introduced on the SP surface, a new absorption appeared at 1648 cm^−1^ for the stretching vibration of the C = O bond of amide [55]. The strengthened absorption at 1230 cm^−1^ was attributable to the stretching vibration of the C–N bond. These results indicated that Pst and PVP had been grafted onto the surface of the SP, and the composite particles Pst-PVP and SP-PVP had been obtained. After immobilizing CRL onto SP-PVP, the band at 3451 cm^−1^ shifted to 3402 cm^−1^, due to the interaction between –OH groups from CRL and SP-PVP. Furthermore, the new absorption at 1537 cm^−1^ appeared for the band of –NH_2_ of CRL. That means the interaction between CRL and SP-PVP carriers had occurred. Furthermore, we prepared PVP-removed SP-Pst by two different methods to immobilize CRL (the relevant data are as seen in the electronic supplementary material). The SP-Pst was prepared by the solution polymerization method with toluene as the solvent, which was assigned as SP/Pst-b. Similarly, the SP-Pst was prepared by the dispersion polymerization method with ethyl alcohol and water as the solvents, which was assigned as SP/Pst-e. As from the electronic supplementary material, figure S4, similarly to a previous report [51], Pst had been grafted onto SP successfully.

For more details, X-ray photoelectron spectroscopy (XPS) was used to investigate the surface compositions of the samples (the relevant data are as seen in the electronic supplementary material). As shown in electronic supplementary material, figure S1, the peaks of Si--OH in three samples SP, SP-PVP, SP-PVP@CRL were all obtained. After PVP was grafted onto SP, the new peaks appeared at the binding energies of 532.1 and 531.8 eV, which are attributed to C–O–C or C = O (seen as electronic supplementary material, figure S2). The results demonstrated that PVP had been grafted onto the surface of the SP, which was consistent with the IR results. The N 1 s spectra of CRL and SP-PVP@CRL are shown in electronic supplementary material, figure S3. For CRL, the peaks at binding energies of 398.6 eV, 399.7 eV and 400.40 eV are attributed to N 1 s, C–N and NH_4_^+^, respectively. After the CRL was immobilized on the supporting materials, the new peak appeared at 401.3 eV for NH_4_^+^, which was obviously shifted to a higher binding energy by 0.9 eV compared to that of CRL, indicating that CRL was bound by electrostatic interaction.

**Figure 3.**
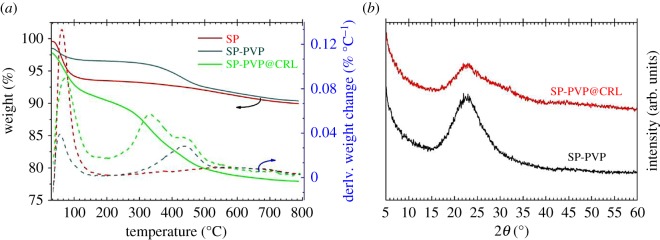
(*a*) The TG profiles of SP, SP-PVP and SP-PVP@CRL; (*b*) the XRD patterns of SP-PVP and SP-PVP @CRL.

The TG analyses of SP, SP-PVP and SP-PVP@CRL are shown in figure 3*a*. As can be seen from figure 3*a*, the mass loss of SP was observed in the range of 50°C to 150°C with the weight loss of water. After PVP is grafted, two weight loss peaks appeared that started at 50°C and 400°C, which are presumed as the weight loss of water and the decomposition of PVP. However, the whole weight loss of the SP-PVP samples was 8.03 wt%, indicating the outstanding thermostability of the SP-PVP materials. Meanwhile, after CRL was loaded on the SP-PVP hybrids, an additional weight loss peak starting at 430°C was observed with a remarkable weight loss, which represents the decomposition of CRL. Furthermore, the powder XRD patterns of SP-PVP before and after adsorption of CRL (seen as figure 3*b*) were measured to explore the influence of CRL immobilization. A strong decrease in XRD peak intensity was observed after CRL adsorption onto the SP-PVP materials, which was responsible for the lower surface crystallinity resulting from CRL loading. However, the peak shape was the same as that of SP-PVP, which indicated the CRL can be immobilized without affecting the structural integrity of the support materials.

**Figure 4.**
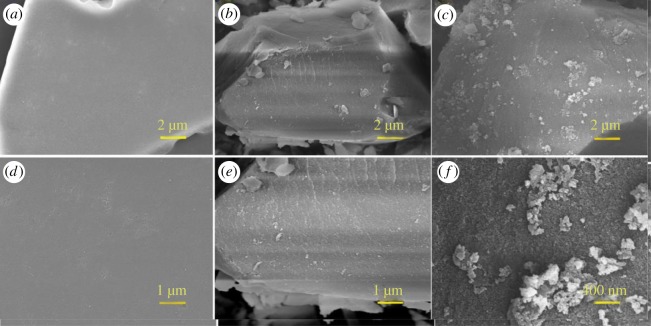
SEM images of (*a*,*d*) SP; (*b*,*e*) SP-PVP; (*c*,*f*) SP-PVP@CRL.

The morphological features of the involved composites were obtained, as shown in figure 4. For pristine SP, typical smooth surface morphologies can be observed (figure 4*a*,*d*). Upon PVP grafting, the SP-PVP products showed significantly visible changes in morphology (figure 4*b*,*e*), by which the surface became rough and irregular because of being covered by the grafted polymer PVP. When CRL was introduced onto the SP-PVP substrate, part of the CRL became grafted to the surface, leading to considerable increase in roughness of the particles’ surface (as seen in the SEM image in figure 4*c*,*f*). This is clear evidence that CRL had been immobilized on the SP-PVP material, which was quite consistent with the FTIR and XPS results.

**Figure 5.**
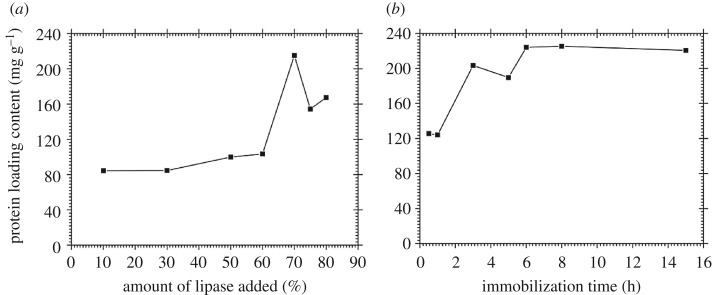
Effect of (*a*) the percentage of lipase added and (*b*) the immobilization time of CRL.

To examine the practical potentiality of SP-PVP, CRL was immobilized on the SP-PVP via physical absorption and the relative effect factors of the process were investigated. The efficiency of immobilization was expressed by the amounts of lipase bound on the supports, which was determined by the Bradford method [39]. The change of protein content with different amounts of lipase is shown in figure 5*a*, where the value of the *x*-axis represents the percentage of lipase content in the materials. According to the data in figure 5*a*, the protein content showed a downward trend when the percentage of lipase added was up to 70%, where the corresponding protein content was 215 mg g^−1^. The downward trend may be caused by the aggregation of CRL during excessive lipase interaction with the SP-PVP supports, which impeded the adsorption of protein. Figure 5*b* shows the effect of immobilizing time on the protein content, where the optimal fixed time is 6 h. After the best immobilization time, the protein content decreased gradually and started to level off. The main reason may be that an appropriate reaction time contributed to coupling the lipase onto the supporting materials. As a result, the optimum condition of immobilizing CRL on SP-PVP materials was obtained: the percentage of lipase was 70% and the immobilization time was 6 h. The bound protein of the immobilized lipase under the optimum condition was 215 mg per gram of support. The effect of pH and temperature on the free and immobilized lipase was also assayed by detecting the activity of the obtained lipase under the optimum condition.

**Figure 6.**
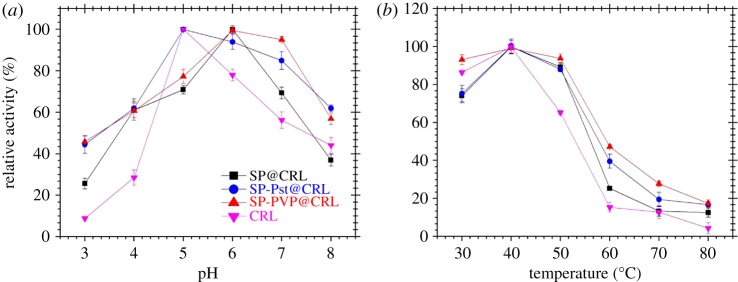
Effect of the (*a*) pH value and (*b*) temperature on the activities of free and three immobilized CRL.

As we know, protein is susceptible to excess acid or alkali, hence selecting an appropriate pH value is quite necessary. For comparison, lipase immobilization with carriers SP@CRL and SP-Pst@CRL of different hydrophobicity was also investigated. As depicted in the results, the activity of SP-PVP@CRL was higher than that of SP@CRL and SP-Pst@CRL, which may be attributed to the amphiphilic PVP on SP-PVP that benefits from the lid-open conformation of lipase. The activity and stability of free and the three immobilized lipases were studied at different pHs in the reaction medium at 37°C (figure 6*a*). For simplicity, we assigned the maximum activity value under optimal conditions as 100% and the activities were expressed as relative activities under other conditions. Compared to the pH endurance of free CRL, all the three immobilized lipases SP@CRL, SP-Pst@CRL and SP-PVP@CRL showed more stability and broader pH scope. The optimum pH shifted from 5.0 for free lipase to 6.0 for SP-PVP@CRL and SP@CRL, which might be due to the effects of the isoelectric point (pI). As we know, the net charge of the protein is negative at a pH higher than and zero at a pH near the isoelectric point. The pI of CRL and the silica surface is around 4.6 and 3.6, respectively, so the net charge of the protein is negative at pH 6.0. Meanwhile, SP and PVP particles, comprising Si--OH and C = O groups, can be considered as weak polyelectrolytes, which would be positive under acidic condition [56]. The amphiphilicity of PVP can combine the buffer effect and hydrophobic interaction for better pH tolerance. For CRL and SP-Pst@CRL, the optimum pH remained at 5.0. It may be explained that the net charge of the protein is zero at pH 5.0, leading to the reduced lateral interactions between the protein molecules [42]. Furthermore, the high hydrophobicity of Pst is lacking in polyelectrolyte groups, which would adjust the local pH around the carriers’ surface. As a result, the CRL on the SP-Pst particle was susceptible to the change of pH. However, the hydrophobic regions of Pst and CRL could well interact with each other for higher stability, leading to a broader pH scope. Moreover, the thermal stability of the free and immobilized CRL was examined in the temperature range from 30 to 80°C. Generally, both of them showed the highest activity at 40°C and SP-PVP@CRL exhibited a much better thermal stability than the free and the other two immobilized ones. In particular, an enhanced thermal stability was presented by which the immobilized CRL retains approximately 20% of its initial activity (in the free CRL nearly no activity was retained). This may be ascribed to the robust conjugation of CRL on the amphiphilic PVP-decorated SP surface, which restricted the structure changes of CRL during the heating process.

**Figure 7.**
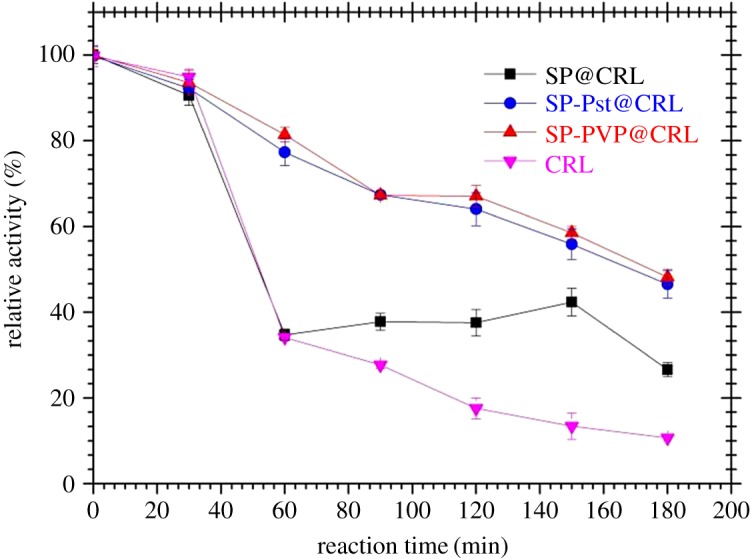
The thermal stabilities of free and three immobilized CRL at 50°C.

The thermal stability of lipase is another critical index for its industrial applications. The residual activity of free and immobilized lipase was measured by hydrolysing *p*-NPP and incubating at 50°C for different time intervals. From figure 7, we can infer that both the free and immobilized CRL exhibit a similar trend, while the relative activities of the three immobilized lipases were considerably higher. The free lipase retained about 10% of its initial activity after 3 h of incubation, while SP-Pst@CRL and SP-PVP@CRL could retain about 50% of their activities after this period of time. For the three immobilized CRL, the hydrophilic SP@CRL lost activity fast and retained 25% of its initial activity, while little difference was observed for the amphiphilic SP-PVP@CRL and hydrophobic SP-Pst@CRL. Upon PVP grafting to the surface of the SP, an amphiphilic interface was formed, which might make the immobilized lipase more adaptable and stable for the change of environment caused by consistent growth of the end-product.

**Figure 8.**
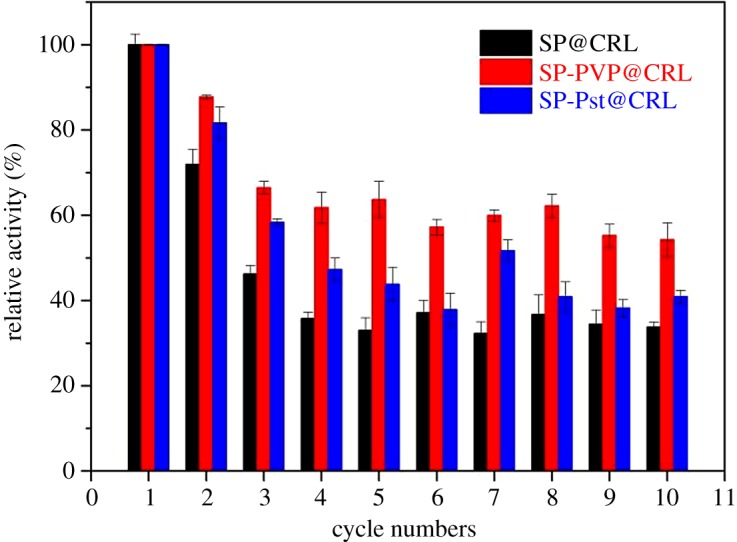
Reusability of the three immobilized lipases.

In addition, the reusability of immobilized lipase is one of its important advantages. To achieve good reusability of lipase, the immobilized CRL was washed with ethanol after one cycle and reintroduced into a next fresh reaction at 37°C. The variations of the activity of the three immobilized CRL after reuse multiple times are shown in figure 8. It can be observed that the activities of all three immobilized lipases decreased with cycles. Considering the fact of weaker physisorption between the lipase and the scaffold, a fraction of lipase leaching was inevitable before reaching the absorption–desorption equilibrium, which may lead to the decrease in activity. According to figure 8, the three obtained immobilized lipases exhibited different cycle performances. The SP-PVP@CRL maintained a higher relative activity at 54% after 10 recycles, while the SP@CRL and SP-Pst@CRL only retain 33% and 40% of their initial activity, respectively. To demonstrate the superiority of amphiphilic PVP functionalization, another two PVP-removed SP-Pst (SP/Pst-b and SP/Pst-e) materials were used as carriers for CRL immobilization. According to the electronic supplementary material, figure S5, SP-PVP@CRL showed better performance that is similar to the above result. From these results, one possible explanation is that the amphiphilic PVP molecule decorated on the surface of the SP may build a protected microenvironment which can enhance the contact between the lipase and the substrate, so as to offer a profitable reaction interface.

## 4. Conclusion

In is work, three support materials, SP, SP-Pst SP-PVP, are designed to immobilize CRL via physical absorption with a maximum protein loading amount of 215 mg g^−1^. The enhanced performance of the three immobilized CRL is expected; however, SP-PVP@CRL exhibits superior performance to that of the free and the other two immobilized CRL. Owing to the high specific surface area and mechanical stability of SP and the amphiphilic characteristic of PVP molecules, CRL immobilized on the PVP-grafted SP support shows higher catalytic activity than that immobilized on the support of hydrophilic SP and hydrophobic SP-Pst. SP@CRL and free CRL lost 75% and 90% of their initial activities, respectively, after 3 h of incubation at 50°C, while SP-Pst@CRL and SP-PVP@CRL retained about 50% of their initial activities. Too hydrophobic or hydrophilic carriers for lipase immobilization are not beneficial for delivering good activity of lipase. In addition, after 10 recycles, SP-PVP@CRL retained 54% of its initial activity, which is higher than that of SP@CRL and SP-Pst@CRL. On the basis of the results from this study, the SP modified by the amphiphilic PVP could be an excellent support for immobilization of lipase, and the immobilized enzyme has promising industrial applications at low cost.

## Supplementary Material

The related XPS spectra of composites.

## Data accessibility

Electronic supplementary material includes models and fitting of the XPS spectra. A complete dataset of measurements is available from Dryad Digital Repository (https://doi.org/10.5061/dryad.2hr3jp7) [57].

## References

[1] Rodrigues RC, Ortiz C, Berenguer-Murcia A, Torresd R, Fernández-Lafuente R 2013 Modifying enzyme activity and selectivity by immobilization. Chem. Soc. Rev. 42, 6290–6307. (doi:10.1039/C2CS35231A) 23059445 10.1039/c2cs35231a

[2] Ge J, Lu DN, Liu ZX, Liu Z 2009 Recent advances in nanostructured biocatalysts. Biochem. Eng. J. 44, 53–59. (doi:10.1016/j.bej.2009.01.002)

[3] Khoobi M, Seyed FM, Zahra A 2014 Synthesis of functionalized polyethylenimine-grafted mesoporous silica spheres and the effect of side arms on lipase immobilization and application. Biochem. Eng. J. 88, 131–141. (doi:10.1016/j.bej.2014.04.009)

[4] Hwang ET, Gu MB 2013 Enzyme stabilization by nano/microsized hybrid materials. Eng. Life Sci. 13, 49–61. (doi:10.1002/elsc.201100225)

[5] Liese A, Hilterhaus L 2013 Evaluation of immobilized enzymes for industrial applications. Chem. Soc. Rev. 42, 6236–6249. (doi:10.1039/c3cs35511j) 23446771 10.1039/c3cs35511j

[6] Iyer PV, Ananthanarayan L 2008 Enzyme stability and stabilization—aqueous and non-aqueous environment. Process Biochem. 43, 1019–1032. (doi:10.1016/j.procbio.2008.06.004)

[7] Sheldon RA, Pelt SV 2013 Enzyme immobilisation in biocatalysis: why, what and how. Chem. Soc. Rev. 42, 6223–6235. (doi:10.1039/C3CS60075K) 23532151 10.1039/c3cs60075k

[8] Qodah ZA, Shannag MA, Busoul MA, Penchev I, Orfali W 2017 Immobilized enzymes bioreactors utilizing a magnetic field: a review. Biochem. Eng. J. 121, 94–106. (doi:10.1016/j.bej.2017.02.003)

[9] Savile CK, Lalonde JJ 2011 Biotechnology for the acceleration of carbon dioxide capture and sequestration. Curr. Opin. Biotechnol. 22, 818–823. (doi:10.1016/j.copbio.2011.06.006) 21737251 10.1016/j.copbio.2011.06.006

[10] Guzik U, Kocurek KH-, Wojcieszyńska D 2014 Immobilization as a strategy for improving enzyme properties—application to oxido reductases. Molecules 19, 8995–9018. (doi:10.3390/molecules19078995) 24979403 10.3390/molecules19078995 PMC6271243

[11] Barbosa O, Ortiz C, Ángel BM, Rodrigues RC, Roberto FL 2015 Strategies for the one-step immobilization-purification of enzymes as industrial biocatalysts. Biotechnol. Adv. 33, 435–456. (doi:10.1016/j.biotechadv.2015.03.006) 25777494 10.1016/j.biotechadv.2015.03.006

[12] Victor MB, Marta MDCV 2015 Structural and functional stabilization of protein entities: state-of-the-art. Adv. Drug Deliver. Rev. 93, 25–41. (doi:10.1016/j.addr.2014.10.005) 10.1016/j.addr.2014.10.005 25312675

[13] Cristina GG, Angel BM, Roberto FL, Rafael CR 2011 Potential of different enzyme immobilization strategies to improve enzyme performance. Adv. Synth. Catal. 353, 2885–2904. (doi:10.1002/adsc.201100534)

[14] Roberto FL 2009 Stabilization of multimeric enzymes: strategies to prevent subunit dissociation. Enzyme Microb. Technol. 45, 405–418. (doi:10.1016/j.enzmictec.2009.08.009)

[15] Cesar M, Jose MP, Gloria FL, Jose MG, Roberto FL 2007 Improvement of enzyme activity, stability and selectivity via immobilization techniques. Enzyme Microb. Technol. 40, 1451–1463. (doi:10.1016/j.enzmictec.2007.01.018)

[16] Secundo F 2013 Conformational changes of enzymes upon immobilisation. Chem. Soc. Rev. 42, 6250–6261. (doi:10.1039/c3cs35495d) 23482973 10.1039/c3cs35495d

[17] Karel H, Roberto FL 2011 Control of protein immobilization: coupling immobilization and site-directed mutagenesis to improve biocatalyst or biosensor performance. Enzyme Microb. Technol. 48, 107–122. (doi:10.1016/j.enzmictec.2010.10.003) 22112819 10.1016/j.enzmictec.2010.10.003

[18] Hamideh V, Hoda JM, Mojgan M, Aydin B, Navideh A, Nahideh J, Shahin N 2016 Application of magnetic nanoparticles in smart enzyme immobilization. Biotechnol. Lett. 38, 223–233. (doi:10.1007/s10529-015-1977-z) 26472272 10.1007/s10529-015-1977-z

[19] Hou C, Qi ZG, Zhu H 2015 Preparation of core-shell magnetic polydopamine/alginate biocomposite for *Candida rugosa* lipase immobilization. Colloids Surf. B 128, 544–551. (doi:10.1016/j.colsurfb.2015.03.007) 10.1016/j.colsurfb.2015.03.007 25784302

[20] Tang DW, Yu SH, Wu WS, Hsieh HY, Tsai YC, Mi FL 2014 Hydrogel microspheres for stabilization of an antioxidant enzyme: effect of emulsion cross-linking of a dual polysaccharide system on the protection of enzyme activity. Colloids Surf. B 113, 59–68. (doi:10.1016/j.colsurfb.2013.09.002) 10.1016/j.colsurfb.2013.09.002 24055882

[21] Katiyar M, Ali A 2015 One-pot lipase entrapment within silica particles to prepare a stable and reusable biocatalyst for transesterification. J. Am. Oil Chem. Soc. 92, 623–632. (doi:10.1007/s11746-015-2630-7)

[22] Zhang S, Shi J, Deng QC, Zheng MM, Wan CY, Zheng C, Li Y, Huang FH 2017 Preparation of carriers based on ZnO nanoparticles decorated on graphene oxide (GO) nanosheets for efficient immobilization of lipase from *Candida rugosa*. Molecules 22, 1205 (doi:10.3390/molecules22071205) 10.3390/molecules22071205 PMC6152098 28753931

[23] Živković LTI, Živković LS, Babić BM, Kokunešoski MJ, Jokić BM, Karadžić IM 2015 Immobilization of *Candida rugosa* lipase by adsorption onto biosafemeso/macroporous silica and zirconia. Biochem. Eng. J. 93, 73–83. (doi:10.1016/j.bej.2014.09.012)

[24] Timin AS, Solomonov AV, Musabirov II, Sergeev SN, Ivanov SP, Rumyantsev EV, Goncharenko A 2014 Immobilization of bovine serum albumin onto porous poly(vinylpyrrolidone)-modified silicas. Ind. Eng. Chem. Res. 53, 13 699–13 710. (doi:10.1021/ie501915f)

[25] Cruz JC, Pfromm PH, Szoszkiewicz R, Rezac ME 2014 Hydrolases on silica surfaces: coverage-activity–molecular property relationships revealed. Process Biochem. 49, 830–839. (doi:10.1016/j.procbio.2014.02.002)

[26] Cao LQ 2005 Carrier-bound immobilized enzymes. Weinheim, Germany: Wiley-VCH.

[27] Hanefeld U, Gardossi L, Manger E 2009 Understanding enzyme immobilisation. Chem. Soc. Rev. 38, 453–468. (doi:10.1039/B711564B) 19169460 10.1039/b711564b

[28] Eliane PC, Alexsandra V, Rosana OH, Denise EM, Jorge LN, Denise MGF, Evelin AM, Roberto FL, de Oliveira D 2016 Nanomaterials for biocatalyst immobilization---state of the art and future trends. RSC Adv. 6, 104675–104692. (doi:10.1039/C6RA22047A).

[29] DiCosimo R, McAuliffe J, Pouloseb AJ, Bohlmann G 2013 Industrial use of immobilized enzymes. Chem. Soc. Rev. 42, 6437–6474. (doi:10.1039/c3cs35506c) 23436023 10.1039/c3cs35506c

[30] Cantone S, Ferrario V, Corici L, Ebert C, Fattor D, Spizzo P, Gardossi L 2013 Effcient immobilisation of industrial biocatalysts: criteria and constraints for the selection of organic polymeric carriers and immobilisation methods. Chem. Soc. Rev. 42, 6262–6276. (doi:10.1039/c3cs35464d) 23525282 10.1039/c3cs35464d

[31] Jesionowski T, Zdarta J, Krajewska B 2014 Enzyme immobilization by adsorption: a review. Adsorption 20, 801–821. (doi:10.1007/s10450-014-9623-y)

[32] Zhang H, Zou D, Shen Y, Gao Y, Zheng X, Zhang XX, Chen XM, Guo YP, Song J 2014 Dominated effect analysis of the channel size of silica support materials on the catalytic performance of immobilized lipase catalysts in the transformation of unrefined waste cooking oil to biodiesel. Bioenerg. Res. 7, 1541–1549. (doi:10.1007/s12155-014-9492-y)

[33] Mahsan K, Isthier C, Cheng J, Mohammad S, Rohollah S, Mehran HR, Jozef K 2014 Immobilization of endo-inulinase on non-porous amino functionalized silica nanoparticles. J. Mol. Catal. B 104, 48–55. (doi:10.1016/j.molcatb.2014.01.025)

[34] Jiang Y, Liu X, Chen Y, Zhou L, He Y, Ma L, Gao J 2014 Pickering emulsion stabilized by lipase-containing periodic meso-porous organo-silica particles: a robust biocatalyst system for biodiesel production. Bioresour. Technol. 153, 278–283. (doi:10.1016/j.biortech.2013.12.001) 24368276 10.1016/j.biortech.2013.12.001

[35] Soozanipour A, Taheri-Kafrani A, Isfahani AL 2015 Covalent attachment of xylanase on functionalized magnetic nanoparticles and determination of its activity and stability. Chem. Eng. J. 270, 235–243. (doi:10.1016/j.cej.2015.02.032)

[36] Wu L, Liu Y, Chi B, Xu Z, Feng X, Li S, Xu H 2015 An innovative method for immobilizing sucrose isomerase on epsilon-poly-L-lysine modified mesoporous TiO_2_. Food Chem. 187, 182–188. (doi:10.1016/j.foodchem.2015.04.072) 25977014 10.1016/j.foodchem.2015.04.072

[37] Zhang T, Xu XL, Jin YN, Wu J, Xu ZK 2014 Immobilization of horseradish peroxidase (HRP) on polyimide nanofibers blending with carbon nanotubes. J. Mol. Catal. B 106, 56–62. (doi:10.1016/j.molcatb.2014.04.015)

[38] Rastian Z, Khodadadi AA, Vahabzadeh F, Bortolini C, Dong MD, Mortazavi Y, Mogharei A, Naseh MV, Guo Z 2014 Facile surface functionalization of multiwalled carbon nanotubes by soft dielectric barrier discharge plasma: generate compatible interface for lipase immobilization. Biochem. Eng J. 90, 16–26. (doi:10.1016/j.bej.2014.05.009)

[39] NurRoyhaila M, Buang NA, Mahat NA, Lok YY, Huyop F, Aboul-Enein HY, Wahab RAB 2015 A facile enzymatic synthesis of geranyl propionate by physically adsorbed *Candida rugosa* lipase onto multi-walled carbon nanotubes. Enzyme Microb. Technol. 72, 49–55. (doi:10.1016/j.enzmictec.2015.02.007) 25837507 10.1016/j.enzmictec.2015.02.007

[40] Kalantari M, Kazemeini M, Arpanaei A 2013 Evaluation of biodiesel production using lipase immobilized on magnetic silica nanocomposite particles of various structures. Biochem. Eng. J. 79, 267–273. (doi:10.1016/j.bej.2013.09.001)

[41] Benaissi K, Hélaine V, Prévot V, Forano C 2011 Efficient immobilization of yeast transketolase on layered double hydroxides and application for ketose synthesis. Adv. Synth. Catal. 353, 1497–1509. (doi:10.1002/adsc.201000925)

[42] Wang JY, Ma CL, Bao YM, Xu PS 2012 Lipase entrapment in protamine-induced bio-zirconia particles: characterization and application to the resolution of (R, S)-1-phenylethanol. Enzyme Microb. Technol. 51, 40–46. (doi:10.1016/j.enzmictec.2012.03.011) 22579389 10.1016/j.enzmictec.2012.03.011

[43] Brzozowski AM et al. 1991 A model for interfacial activation in lipases from the structure of a fungal lipase-inhibitor complex. Nature 351, 491–494. (doi:10.1038/351491a0) 2046751 10.1038/351491a0

[44] Cygler M, Schrag JD 1999 Structure and conformational flexibility of *Candida rugosa* lipase. Biochim. Biophys. Acta 1441, 205–214. (doi:10.1016/S1388-1981(99)00152-3) 10570248 10.1016/s1388-1981(99)00152-3

[45] Schmid RD, Verger R 1998 Lipases interfical enzymes with attractive applications. Angew. Chem. Int. Ed. 37, 1608–1633. (doi:10.1002/(SICI)1521-3773(19980703)37:12<1608::AID-ANIE1608>3.0.CO;2-V) 10.1002/(SICI)1521-3773(19980703)37:12<1608::AID-ANIE1608>3.0.CO;2-V 29711530

[46] Verger R 1997 Interfical activation of lipase: facts and artifacts. Tends Biotechnol. 15, 32–38.

[47] Evelin AM, José CSS, Denise MGF, Nazzoly R, Roberto FL 2015 Immobilization of lipases on hydrophobic supports involves the open form of the enzyme. Enzyme. Microb. Technol. 71, 53–57. (doi:10.1016/j.enzmictec.2015.02.001) 25765310 10.1016/j.enzmictec.2015.02.001

[48] Galina AK, Anatoly BB, Larisa VP, Aleksey LM, Nina AR, Sergey IM, Vladimir LK 2013 Immobilization of recombinant *E. coli* thermostable lipase by entrapment inside silica xerogel and nanocarbon-in-silica composites. J. Mol. Catal. B Enzym. 98, 78–86. (doi:10.1016/j.molcatb.2013.09.022)

[49] Jochems P, Satyawali Y, Dielsa L, Dejonghe W 2011 Enzyme immobilization on/in polymeric membranes: status, challenges and perspectives in biocatalytic membrane reactors (BMRs). Green Chem. 13, 1609–1623. (doi:10.1039/c1gc15178a)

[50] Yu WH, Tong DS, Fang M, Shao P, Zhou CH 2015 Immobilization of *Candida rugosa* lipase on MSU-H type mesoporous silica for selective esterification of conjugated linoleic acid isomers with ethanol. J. Mol. Catal. B Enzym. 111, 43–50. (doi:10.1016/j.molcatb.2014.11.003)

[51] Gao BJ, Wang RX, Jiu HF, Kong DL 2010 A comparative study on effects of two kinds of polymerization methods on grafting of polymer onto silica surface. J. Appl. Polym. Sci. 102, 5808–5817. (doi:10.1002/app.24819)

[52] Bourgeat-Lami E, Lang J 1998 Encapsulation of inorganic particles by dispersion polymerization in polar media 1. Silica nanoparticles encapsulated by polystyrene. J. Colloid Interface Sci. 197, 293–308. (doi:10.1006/jcis.1997.5265) 9466871 10.1006/jcis.1997.5265

[53] Bourgeat-Lami E, Lang J 1999 Encapsulation of inorganic particles by dispersion polymerization in polar media 2. Effect of silica size and concentration on the morphology of silica–polystyrene composite particles. J. Colloid Interface Sci. 210, 281–289. (doi:10.1006/jcis.1998.5939) 9929415 10.1006/jcis.1998.5939

[54] Bradford MM 1976 A rapid and sensitive method of the quantitation for microgram quantities of protein utilizing the principle of protein-dye binding. Anal. Biochem. 72, 248–254. (doi:10.1016/0003-2697(76)90527-3) 942051 10.1016/0003-2697(76)90527-3

[55] Chan CK, Chu IM, Ou CF, Lin Y-W 2004 Interfacial interactions and their influence to phase behavior in poly(vinyl pyrrolidone)/silica hybrid materials prepared by sol–gel process. Mater. Lett. 58, 2243–2247. (doi:10.1016/j.matlet.2004.01.026)

[56] Shi J, Wang X, Zhang S, Tang L, Jiang Z 2016 Enzyme-conjugated ZIF-8 particles as efficient and stable Pickering interfacial biocatalysts for biphasic biocatalysis. J. Mater. Chem. B 4, 2654–2661. (doi:10.1039/C6TB00104A) 10.1039/c6tb00104a 32263289

[57] Zhang S, Deng Q, Li Y, Zheng M, Wan C, Zheng C, Tang H, Huang F, Shi J 2018 Data from: Novel amphiphilic polyvinylpyrrolidone functionalized silicone particles as carrier for low-cost lipase immobilization Dryad Digital Repository. (https://dx.doi.org/10.5061/dryad.2hr3jp7) 10.1098/rsos.172368 PMC6030335 30110464

