# Supplementary material for: Novel amphiphilic polyvinylpyrrolidone functionalized silicone particles as carrier for low-cost lipase immobilization
Source: R Soc Open Sci. 2018 Jun 13;5(6):172368. doi: 10.1098/rsos.172368 (PMC6030335; doi:10.1098/rsos.172368)
Supplement: The related XPS spectra of composites. [file rsos172368supp1.docx]

**Supporting Infromation**

**Novel amphiphilic polyvinylpyrrolidone (PVP) functionalized silicone particles as carrier for low cost lipase immobilization**

Shan Zhang, Qianchun Deng, Ya Li, Mingming Zheng, Chuyun Wan, Chang Zheng, Hu Tang, Fenghong Huang* and Jie Shi*

Hubei Key Laboratory of Lipid Chemistry and Nutrition, Oil Crops and Lipids Process Technology National & Local Joint Engineering Laboratory, Key Laboratory of Oilseeds Processing, Ministry of Agriculture, Oil Crops Research Institute, Chinese Academy of Agricultural Sciences, Wuhan 430062, China.


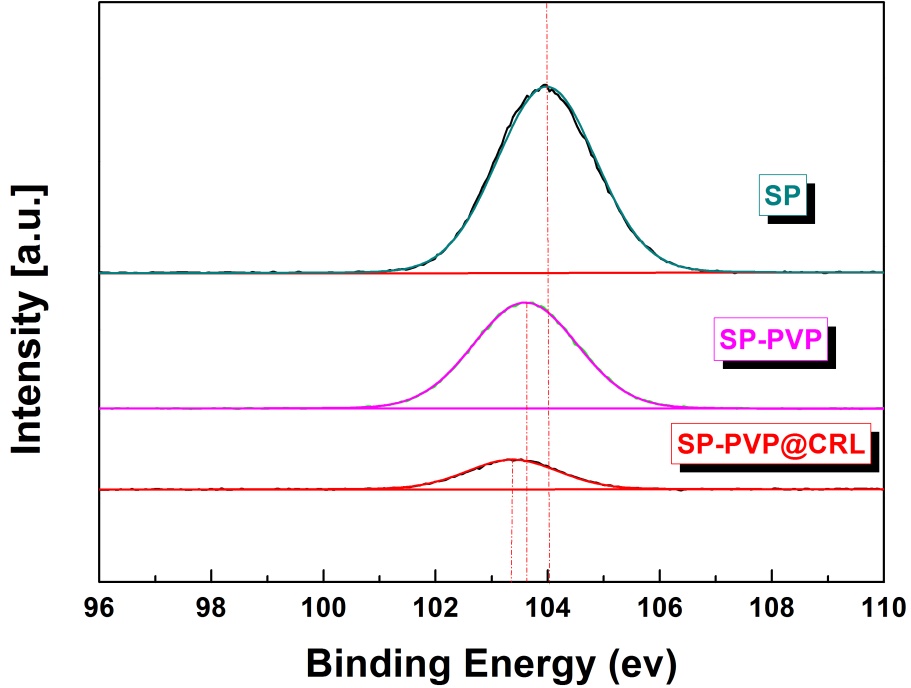


**Figure S1.** XPS spectra of Si spectra of PVP-SP, PVP-SP@CRL.

**Table S1.** The data of the each peak for the supporting materials.

| **Sample** | **Peak 1** |
| --- | --- |
| SP | 103.98 |
| PVP-SP | 103.60 |
| PVP-SP@CRL | 103.37 |
|  | Si-OH |


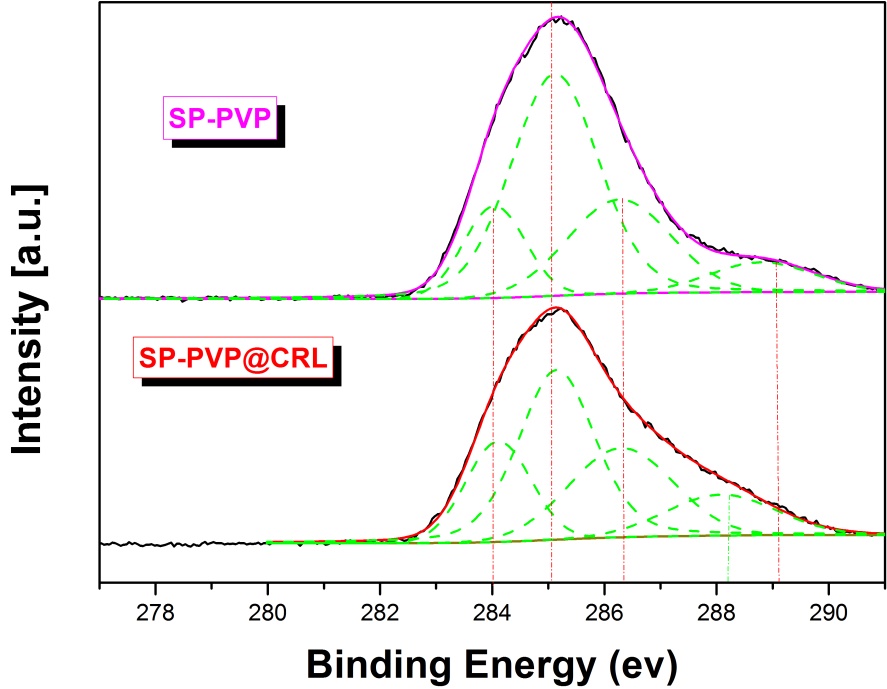


**Figure S2.** XPS spectra of C 1s spectra of PVP-SP, PVP-SP@CRL

**Table S2.** The data of the each peak for the supporting materials

| **Sample** | **Peak 1** | **Peak 2** | **Peak 3** | **Peak 4** |
| --- | --- | --- | --- | --- |
| PVP-SP | 288.80 | 286.27 | 285.12 | 284.04 |
| PVP-SP@CRL | 288.08 | 286.30 | 285.15 | 284.11 |
|  | C=O | C-OH/C-O-C | C-N | （CH_2_）_n_ |


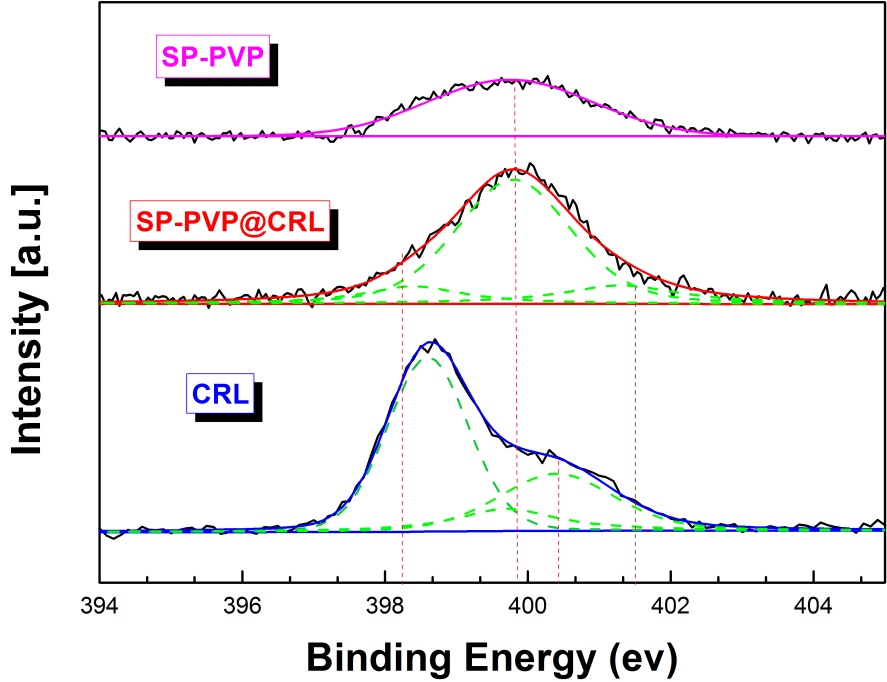


**Figure S3.** XPS spectra of N 1s spectra of PVP-SP, PVP-SP@CRL.

**Table S3.** The data of the each peak for the supporting materials.

| **Sample** | **Peak 1** | **Peak 2** | **Peak 3** |
| --- | --- | --- | --- |
| PVP-SP | / | 399.75 | / |
| SP-PVP@CRL | 401.30 | 399.80 | 398.40 |
| CRL | 400.40 | 399.70 | 398.60 |
|  | NH_4_ | CN | N 1S |


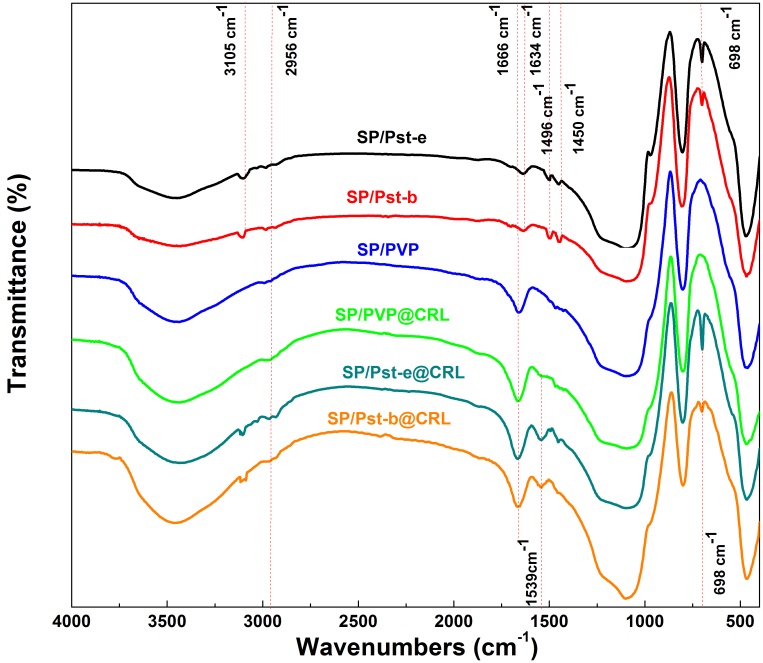


Fig S4. The FTIR spectra of SP/PVP, SP/Pst-b, SP/Pst-e, SP/Pst-b@CRL, SP/Pst-e@CRL and SP-PVP@CRL.


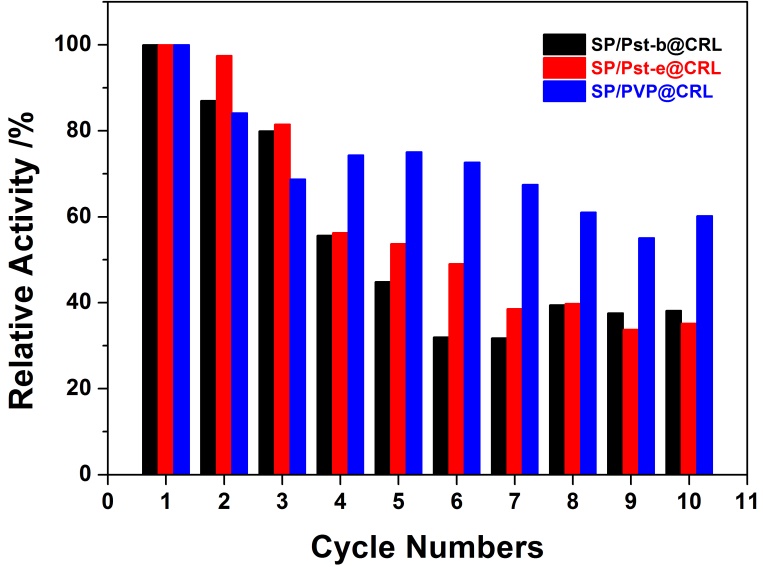


Fig S5. Reusability of the three immobilization lipases.

The method ^[51-53]^ of prepare Pst-grafted SP was described below:

1. Dispersion polymerization

3 g MPS-SP were dispersed in 200 g ethanol/water mixture (94.5/5.5, wt/wt) and the mixture was treated with ultrasonic for 30 min. Then 2 g styrene and 0.2 g AIBN was added slowly and the reaction was carried out at 70 °C under N_2_ atmosphere for 12 h. After the termination of polymerization, the mixture was ﬁltrated and washed with ethanol more than 5 times. Finally the products were dried under vacuum to obtain the pale grays SP/Pst-e materials.

1. Solution polymerization

Solvent toluene, MPS-SiO_2_, and styrene were added in turn into a three-necked flask equipped with a water condenser and N_2_ was purged for 30 min so as to eliminate air. In the inert atmosphere of N_2_, the content of flask was first agitated for 1h to disperse MPS-SiO_2_ fully, then the temperature was increased up to 80 ^o^C, initiator AIBN was introduced, and the reaction was carried out for 7 h. After the termination of polymerization, the mixture was filtrated, and the product was extracted with toluene in soxhlet extractor for 20 h, and then washed with ethanol. Finally the product was dried under vacuum
